# Neuronal Effects of Listening to Entrainment Music Versus Preferred Music in Patients With Chronic Cancer Pain as Measured via EEG and LORETA Imaging

**DOI:** 10.3389/fpsyg.2021.588788

**Published:** 2021-02-25

**Authors:** Andrea McGraw Hunt, Jörg Fachner, Rachel Clark-Vetri, Robert B. Raffa, Carrie Rupnow-Kidd, Clemens Maidhof, Cheryl Dileo

**Affiliations:** ^1^Department of Music, Rowan University, Glassboro, NJ, United States; ^2^Cambridge Institute for Music Therapy Research, Anglia Ruskin University, Cambridge, United Kingdom; ^3^Josef Ressel Centre for Personalised Music Therapy, IMC University of Applied Sciences Krems, Krems an der Donau, Austria; ^4^Department of Pharmacy Practice, School of Pharmacy, Temple University, Philadelphia, PA, United States; ^5^Department of Pharmaceutical Sciences, School of Pharmacy, Temple University, Philadelphia, PA, United States; ^6^College of Pharmacy, University of Arizona, Tuscon, AZ, United States; ^7^South Woods State Prison, Rutgers University Behavioral Health Care, Bridgeton, NJ, United States; ^8^Department of Music Education and Therapy, Boyer College of Music and Dance, Temple University, Philadelphia, PA, United States

**Keywords:** music therapy, EEG, LORETA (Low Resolution Electromagnetic Tomography), case study, chronic pain, cancer, pain

## Abstract

Previous studies examining EEG and LORETA in patients with chronic pain discovered an overactivation of high theta (6–9 Hz) and low beta (12–16 Hz) power in central regions. MEG studies with healthy subjects correlating evoked nociception ratings and source localization described delta and gamma changes according to two music interventions. Using similar music conditions with chronic pain patients, we examined EEG in response to two different music interventions for pain. To study this process in-depth we conducted a mixed-methods case study approach, based on three clinical cases. Effectiveness of personalized music therapy improvisations (entrainment music – EM) versus preferred music on chronic pain was examined with 16 participants. Three patients were randomly selected for follow-up EEG sessions three months post-intervention, where they listened to recordings of the music from the interventions provided during the research. To test the difference of EM versus preferred music, recordings were presented in a block design: silence, their own composed EM (depicting both “pain” and “healing”), preferred (commercially available) music, and a non-participant’s EM as a control. Participants rated their pain before and after the EEG on a 1–10 scale. We conducted a detailed single case analysis to compare all conditions, as well as a group comparison of entrainment-healing condition versus preferred music condition. Power spectrum and according LORETA distributions focused on expected changes in delta, theta, beta, and gamma frequencies, particularly in sensory-motor and central regions. Intentional moment-by-moment attention on the sounds/music rather than on pain and decreased awareness of pain was experienced from one participant. Corresponding EEG analysis showed accompanying power changes in sensory-motor regions and LORETA projection pointed to insula-related changes during entrainment-pain music. LORETA also indicated involvement of visual-spatial, motor, and language/music improvisation processing in response to his personalized EM which may reflect active recollection of creating the EM. Group-wide analysis showed common brain responses to personalized entrainment-healing music in theta and low beta range in right pre- and post-central gyrus. We observed somatosensory changes consistent with processing pain during entrainment-healing music that were not seen during preferred music. These results may depict top–down neural processes associated with active coping for pain.

## Introduction

Chronic pain is a complex, multi-dimensional phenomenon that, by its nature, is difficult to effectively treat. This is particularly true in the United States where chronic pain affects more than 100 million per year and has a profound effect on quality of life partly due to the nation’s healthcare and medical systems. The economic cost of pain in the U.S. ranges from $560–$635 billion annually including up to $336 billion due to lost work productivity [Institute of Medicine (US) Committee on Advancing Pain Research, Care, and Education, 2011]. Chronic pain also affects morbidity, immune function, sleep, cognition, eating, mobility, psychosocial state and behaviors, and overall functioning. For this reason, chronic pain is often regarded as a disease in itself, due to its long-term physiological and psychological effects that require unique assessment and treatment. Opioid analgesic pain relievers continue to be the cornerstone therapy for the treatment of moderate to severe pain in the U.S. (Dowell et al., 2016). Between 1999 and 2016, the number of prescriptions for opioids in the US has quadrupled [Substance Abuse, and Mental Health Services Administration (US) Office of the Surgeon General (US), 2016] making opioid analgesics the most prescribed class of medications in the nation at more than 289 million prescriptions per year (Volkow et al., 2011; Levy et al., 2015). The increase in prescriptions for opioid pain relievers has been accompanied by dramatic increase in misuse and by a more than 200% increase in the number of emergency department visits (Crane, 2013). From 1999 to 2015, more than 183,000 people have died in the U.S. from overdoses related to prescription opioids. Complicating the treatment of chronic pain, patients present with very diverse responses to similar physical causes of pain and treatments for pain (Turk and Monarch, 2002). Therefore, chronic pain treatment requires an individualized approach, with increased potential for non-pharmacological interventions.

Pain has been defined as not only a physical sensation, but also an affective and psychological phenomenon, that can be shaped by life experience (International Association for the Study of Pain, 2017). Music therapy interventions designed for pain management seem to be well-suited to address such needs because clinicians can address these biopsychosocial domains in an integrated, synergistic way (Bernatzky et al., 2011). Thus, health practitioners have long utilized music interventions for alleviating pain, and systematic reviews and meta-analyses of the literature over the past 15 years have found that music interventions reduce pain and reduce requirements for morphine-like analgesics (Cepeda et al., 2006; Dileo, 2006; Bradt et al., 2016a; Lee, 2016). Subsequent research adds to this evidence base, demonstrating that listening to music reduces both acute and chronic pain (Nilsson, 2008; Guétin et al., 2012; Roy et al., 2012; Garza-Villarreal et al., 2014; Korhan et al., 2014).

Most research regarding music intervention for pain utilizes listening to commercially recorded music chosen by the participant with available selections containing relaxing, sedative qualities, and often administered by medical personnel such as nursing staff. Dileo (1999) has referred to such applications as *music medicine*, in comparison to *music therapy* which requires an interpersonal, music-based relationship with a music therapist. A contrasting music therapy intervention called *Entrainment* (Dileo and Bradt, 1999) utilizes live music as specified by the participant with the assistance of a trained music therapist; based upon the phenomenon of rhythmic synchrony of oscillations observed in physics, this music is designed to “entrain” with the participant’s internal experience of the pain and then shift to a healing sensation or experience. A main theoretical premise for these two phases involves the therapist’s empathetic relationship with both the client and his/her pain, through the process of an extensive verbal interview and musical experimentation and improvisation between client and therapist to imagine the sound of the pain through various musical elements including instrument choices, tone color, dynamics, pulse, rhythm, and/or pitch. For example, Metzner (2012) described how a client chose to scrape a metal rod on the edge of a cymbal to depict a sharp, icy sensation of her pain perception. Through a similar process of interview and sound exploration, the client and therapist then develop a depiction of healing music. Metzner (2012) also described how the same client chose the sound of an ocean drum as her healing music to connect with a positive memory of a visit to the ocean and a rainstorm. After assisting the participant in objectively describing the pain through music, the therapist then resonates with the participant’s pain and healing experience by playing the participant-created music while the participant is in a relaxed, aware state. Simultaneously, the participant directs the therapist’s playing in terms of volume, pace, and intensity through non-verbal cues–for example, if the pain has a pulse or rhythm, sharpness or dullness, the participant indicates these qualities to the therapist as the therapist plays. Thus the therapist validates the participant’s pain and healing experience and provides unique support for the participant in coping with the pain. Metzner (2012) refers to this intervention by its German term *Musik-imaginative Schmerzbehandlung* (“music-imaginative pain treatment”) and describes how the intervention incorporates the subjective experience of pain sensation and perception, followed by the intersubjective phenomena of pain description and expression. Because the purpose of this intervention is to increase focus on the physical experience of pain and healing as a means of promoting increased control over pain perception, Hauck et al. (2013) describe Entrainment as the use of music as a means of active coping. We would add that the participant’s recollection of the music improvised in an entrainment session would also involve the memory of the shared experience of pain and healing with the music therapist, providing another essential component of “active coping” that would last beyond the intervention itself. Several studies have demonstrated the effectiveness of Entrainment on pain perception (Rider, 1985; Schwoebel et al., 2002; Bradt, 2010; Hauck et al., 2013).

Despite the clinical evidence of music’s effect on pain perception, there are inconsistencies across studies, which Howlin and Rooney (2020) attribute to a lack of attention to prospective cognitive mechanisms for music interventions for pain. Furthermore, there is no published evidence of neurological correlates of music interventions for chronic pain. Preliminary research shows a possible relationship between musical reward (both listening and singing) and activation of the nucleus accumbens (NAc) as well as midbrain nuclei found to regulate morphine analgesia and descending inhibition of pain (Chanda and Levitin, 2013). Based on this knowledge, music therapists have theorized that music can reduce chronic pain perception by influencing activity in these brain regions while simultaneously modulating mood and cognitive reactions to the pain in the prefrontal cortex and the limbic and paralimbic regions (Bradt et al., 2016b).

Studies on neural oscillations in patients with chronic pain have produced mixed findings. Two recent reviews (Pinheiro et al., 2016; Ploner et al., 2017) into neuronal responses to pain highlight a common theme of overactivation of theta frequencies linked to thalamocortical pain networks. Two such studies include Stern et al. (2006) and Sarnthein et al. (2006) which examined resting-state neuronal oscillations in patients with neuropathic pain (versus healthy controls) to discover an overactivation of high theta (6–9 Hz) and low beta (12–16 Hz) power in central regions, including insula and insular cortices. In their review, Pinheiro et al. (2016) also reported increased alpha power in chronic pain patients at rest, and Ploner et al. (2017) noted a pattern of increased beta oscillations in frontal regions. Both Pinheiro et al. (2016) and Ploner et al. (2017) note that neuronal behavior in chronic pain patients occurs in an extended network of regions, including somatosensory, insular, cingulate, and prefrontal cortices, as well as the thalamus, subcortical areas, and the brainstem. This indicates that pain perception and processing involves a dynamic network of activity across brain regions, rather than localized activity. Whereas results in the reviewed studies are inconsistent, nevertheless changes in theta and beta oscillations and slowing of peak frequency seem to be common observations in patients with chronic pain (Ploner et al., 2017).

In a groundbreaking MEG study of responses to induced pain during listening to preferred music versus recordings of personalized entrainment music, Hauck et al. (2013) found that preferred music led to decreased delta power in the cingulate gyrus, whereas entrainment music led to changes in gamma power in somatosensory regions. Specifically, the ‘pain’ segment of the entrainment music led to increased gamma power, and the ‘healing’ music led to decreased gamma power. Hauck et al. (2013) hypothesized that the different attentional demands toward or away from pain could influence the perception of pain in different ways, and these demands would be reflected in neuronal oscillations. The increased gamma activity during the pain portion of the entrainment condition could reflect top–down neural processing, involving attentional demands to modulate the perception of pain as controllable. In contrast, the decreased delta activity in the MEG during the preferred music condition reflects known responses to distraction; this indicated that participants shifted their attention away from the pain and toward the preferred music.

Lu et al. (2019) reported lower ratings on pain unpleasantness while listening to preferred music and ‘reduced magnitude of prestimulus EEG oscillations’ (p. 3337) in lower frequency ranges (4–15 Hz) compared to white noise or silence. Ploner et al. (2017) discuss the mechanisms of top–down and bottom–up processes as studied in research of evoked, or phasic, pain. In studies of intracranial recordings, researchers found that when participants attended to pain stimuli, the medial prefrontal cortex exerted causal influences on the primary sensorimotor cortex; the causal influences were reversed when participants were distracted from pain. As with Hauck et al. (2013), these dynamics relate to evoked pain perception rather than chronic pain, but these mechanisms may also be relevant to chronic pain because of the different attentional demands of preferred music versus entrainment music interventions.

**FIGURE 1:**
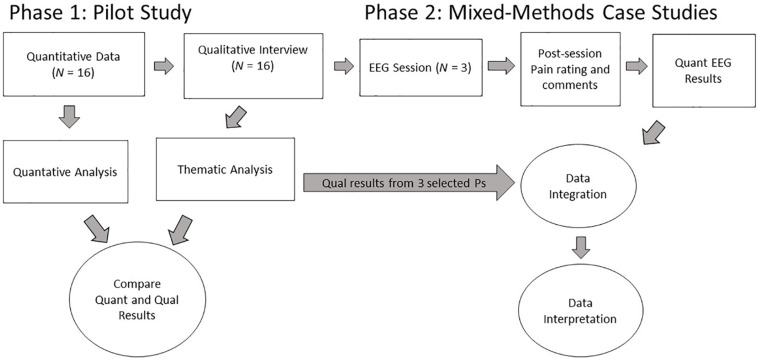
Procedural diagram for Phases 1 and 2 of the current study. This report focuses on the data and analysis of Phase 2.

Therefore, to obtain preliminary data toward understanding the effects of preferred versus entrainment music interventions on chronic pain *in vivo*, we conducted EEG case studies with three randomly selected participants out of the 16 participants (see Figure 1) to compare neuronal responses to entrainment (requiring careful attention to the nature and parameters of the pain, including its musical features) and preferred (using pre-recorded commercial music selected by participants) listening conditions. Pinheiro et al.’s (2016) systematic review suggested that quantitative EEG ‘could be considered as a simple and objective tool for the study of brain mechanisms involved in chronic pain’ (p. 1), therefore we applied an objectivist case study design (Ridder and Fachner, 2016) to study proposed changes of brain processes of music therapy interventions in-depth with three chronic pain patients utilizing quantitative EEG (qEEG).

As in Hauck et al. (2013), we were interested in how the two segments of the entrainment music, namely the pain and the healing music (defined below), differed in their representation in the EEG. As this music was based on an interactive process between a therapist and the client, this music was individualized and consequently, these interventions had different durations. To control for the personal connection to this music we also asked the participants to listen to the entrainment music of another participant.

Previous research on entrainment music indicated that personalized music has a strong impact on patient’s pain perception. Based on the EEG literature on pain processing, we developed four propositions for the corresponding EEG frequency ranges and the related qualitative data. Expecting that personalized music has a stronger impact on pain processing our propositions for the planned EEG comparisons and the related qualitative data were:

(1) We expected differences in absolute power at theta, beta, and/or gamma frequencies according to the personalization of the segment depicting “pain” in the entrainment music (comparing personalized pain music to another participant’s pain music).
(2) We expected differences in absolute power at theta, beta, and/or gamma range according to the personalization of the segment depicting “healing” in the entrainment music (comparing personalized healing music to another participant’s healing music).
(3) We expected differences in theta, beta, and/or gamma frequencies when comparing post-entrainment resting state with baseline resting state.
(4) We expected differences in absolute power at delta, theta, beta, and/or gamma frequencies when comparing participants’ personalized healing music segments from the entrainment music to their individualized preferred music condition.

Given that there is no evidence of one-to-one correspondence of pain processing at any brain region (Ploner et al., 2017), we first focused our examination on power changes in different frequency bands and then utilized Low-Resolution Brain Electric Tomography (LORETA) (Pascual-Marqui et al., 1994) for absolute power differences at those frequencies to determine localized activity.

## Materials and Methods

### Design

The present study was the second phase of a two-phase research project. Phase 1 (Dileo et al., in preparation) was a pilot project examining the effects of two different music interventions on chronic pain in cancer patients, whereas Phase 2 (the present study) involved a subset of Phase 1 participants to examine their neuronal responses to recordings of the music interventions from Phase 1. Figure 1 depicts the design of the entire research project from which the present data and propositions were derived in Phase 2.

#### Summary of Phase 1

In Phase 1, participants individually met with a music therapist for two separate sessions. In one session, the therapist offered participants a selection of commercially recorded instrumental music from which they could select music that matched their preferences given their current physical and psychological state. With the assistance of the music therapist, the participants listened to this music for the duration each of them individually deemed necessary to alleviate their pain (“preferred music” condition). In the other session, participants worked with the music therapist to select instruments and musical sounds to create music that, first, portrayed and matched the sensory experience of their pain, and then, second, portrayed a healing experience (“entrainment music” condition, with a “pain” component and a “healing” component). As part of the protocol for implementing the Entrainment intervention (Dileo and Bradt, 1999), participants directed the music therapist to start and end each component of the improvised music. The therapist audio recorded both the control and experimental sessions to monitor treatment fidelity and for use in the second phase of the study. At the end of Phase 1, participants were interviewed during their final visit to gain their perspective about the two interventions’ effects on their pain. The interviewer conducted the interview using a phenomenological approach, asking open-ended questions to understand the participants’ experiences during each intervention, including their own understanding of the similarities and differences of their experience in each condition. The interviews were audio recorded and transcribed. Sentences or phrases from the transcriptions were coded according to their essences, and these codes were sorted into categories (Saldana, 2015). Themes emerged within and across each music condition, for example, “Relaxation and body responses,” “Connection with music,” “Negative reactions,” “Positive reactions,” “Time,” etc. The full report of these qualitative results from Phase 1 will be detailed in Dileo et al. (in preparation).

#### Summary of Phase 2

We selected three participants from Phase 1 to participate in Phase 2. These participants underwent individual EEG sessions under several listening conditions, including music from their sessions in Phase 1. Phase 2, the present study, incorporates both quantitative and qualitative data in the forms of quantitative EEG and qualitative interviews. Figure 1 shows the points of data collection for each type and set of data in the overall project. The thematic results of these participants’ qualitative interviews from Phase 1 were integrated into the results from the quantitative EEG outcomes and interpreted according to our propositions. Temple University’s Institutional Review Board approved both phases of the research study.

### Participants

We recruited potential participants for Phase 1 from the pain clinic at an outpatient cancer center at a major urban hospital. Eighteen participants consented to participate and, of these, 16 completed the full protocol of Phase 1. Twelve weeks after Phase 1 ended, we randomly selected three right-handed participants who still met the study criteria to participate in Phase 2. Each participant in Phase 1 had been assigned a participant number for data management and tracking. Eighteen participants enrolled in Phase 1, and two participants withdrew prior to data collection. We used a web-based random number generator to select three numbers between 1 and 18, eliminating the participant numbers of the participants who had withdrawn from the study. We then contacted the participants assigned to those numbers selected by the application.

These participants then gave their informed consent to this second phase. Table 1 shows the participants’ demographic information and their pain and analgesic history entering Phase 1. We obtained general information about the type, dosage, and frequency of each participants’ medications and did not ask participants to refrain from taking any medication for the EEG studies so as to keep their pain under control. Because participants served as their own controls, and the effects of all the participants’ medications on their EEGs would be difficult to isolate, we proceeded with the understanding that this project would provide preliminary data toward a study with more controls on medication effects on EEG.

**TABLE 1:** Participant information.

**Participant^a^**	**Age range**	**Tumor type^b^**	**Treatment status**	**Pain location**	**Pain duration**	**Pain type^c^**	**Avg narcotic dose/day**	**Avg breakthrough doses/day**
Darryl	55–60	HT	Active	Legs, lower back	>1 year	N	30–100 mg	3–4
Will	70–75	ST	Observation	Head, jaw	>1 year	S, N	>100 mg	1–2
Carolyn	60–65	ST	Observation	Upper legs, knees	>1 year	S, N	>30 mg	3–4

*^*a*^The participants’ names have been changed to pseudonyms to maintain confidentiality and for ease of reference.*

*^*b*^Tumor types: ST, solid tumor; HT, hematologic tumor.*

*^*c*^Pain types: N, neuropathic; S, somatic.*

### EEG Conditions, Music Presentation, and Pain Assessment

**FIGURE 2:**
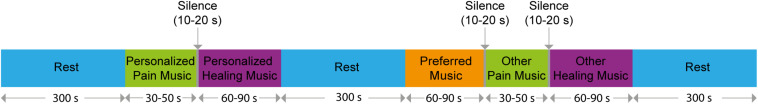
Block design of stimulus conditions (presented in order from left to right). Resting state, eyes closed; Personalized Pain Music (Entrainment) Recording; Personalized Healing Music (Entrainment) Recording; Post Personalized Entrainment Music Rest Period; Preferred Music; Control Condition: Other Pain (Entrainment) Music; Control Condition: Other Healing (Entrainment) Music; Post Other Entrainment Music Rest Period. Note: 10–20 s of silence for transitions between each Pain/Healing recording, and between Preferred Music and Other Pain (Entrainment) Music.

Given the small physical space of the EEG lab, the logistics of providing live entrainment music during EEG acquisition would have been very challenging and limited. Because the entrainment sessions were recorded during Phase 1 to assure treatment fidelity, we determined using these recordings would provide a wider array of instrumentation and musical potential than using very limited, live instrumentation inside the lab. Therefore, during each EEG session, we presented a recorded series of music conditions, customized for each participant, in a block design (Figure 2), which would also provide uniformity in auditory presentation. The block design included segments of audio recordings collected from the individual participants’ entrainment and preferred music therapy sessions from Phase 1 of the study. We also presented a recording of an entrainment music session of a fourth participant (pain and healing) to compare with each participant’s entrainment music as a control condition. These recordings were obtained using an omnidirectional microphone and Garageband software to ensure high-quality audio recordings in the clinical setting (in Phase 1). The duration of each music condition in the block design was determined by the length of music available from each participant’s session. Because the improvised music from the entrainment sessions was shorter in duration than the music selections from the preferred music sessions, the entrainment segments were shorter, utilizing a minimum of 30 s of music for each of the pain and healing conditions. Though the preferred music sessions in Phase 1 of the study were 10 or more minutes long for all the participants, we chose to use between 60 and 90 s of each participant’s music in the block design in the present study, so it would not be significantly longer than the entrainment segments. The resting conditions following the entrainment music conditions were 5 min long. The music therapy clinician from the first phase of the study selected the most salient segments of music from each participant’s sessions for each music condition and created a playlist of the music and silent segments in the appropriate sequence for the block design. The clinician also included 10–20 s of silence in between each music transition (e.g., from both presentations of entrainment “pain” to “healing” music, and between “Preferred” and “Other Pain” conditions) to most closely replicate a realistic clinical scenario. Regarding presentation order, we kept the “pain” and “healing” segments together from their respective entrainment sessions to again ensure the presentation was as close to a real clinical situation as possible. Because we were working with only three cases, there was little need to randomize the presentation order. The length of the resulting block designs ranged from 19 to 21.5 min for each participant.

Immediately prior to and after the EEG study, the music therapist asked each participant to rate the severity of his/her pain on a scale of 1–10 and to answer focused interview questions inquiring about each participant’s experience during each condition of the EEG session, as well as what part of the session, if any, was most helpful for the participant’s pain. The music therapist notated the participants’ responses in their own words. Because of the brief nature of these interviews, the responses stood on their own without need for additional coding or analysis and served to provide context for interpreting the quantitative data (EEG) in Phase 2. See Appendix A for a description of each participant’s music conditions.

### EEG Assessment

#### Recording Technology

We recorded EEG with 21 scalp (Ag-AgCl) electrodes using a NicoletOne station with a v44 amplifier, integrated Video EEG, and NicVue recording software v.3.0.6 (Nicolet Biomedical, Madison, WI, United States). Electrodes were attached to the scalp according to the international 10/20 system at Fp1, Fp2, F3, F4, C3, C4, P3, P4, O1, O2, F7, F8, T3, T4, T5, T6, Fz, Cz, and Pz. EEG and artifact control signals were amplified with a band pass filter of 0.053–55 Hz with sensitivity set at 7 mV. Impedance was under 5 ohms with a sample rate of 256 Hz.

We recorded EOG from two additional electrodes placed at the outer canthi of each eye and ECG from one chest electrode. Two electrodes on each earlobe served as reference, with an additional pair of electrodes placed on the mastoids for ground.

#### Procedure and Recording Setting

The EEG sessions took place in the outpatient EEG lab at the same hospital which housed the participants’ cancer clinic. The individual sessions took place after normal clinic hours in the late afternoon to limit noise disturbance from outside the examination room. We connected each participant to the acquisition station and checked conduction and impedance. Once setup was complete, the music therapist then briefed each participant on what would occur during the EEG study. Participants were instructed to rest with eyes closed, listen to each music segment, and focus on the effects of the different segments on their pain. The therapist then started the session by playing the CD created for each respective participant on a Sony portable stereo placed in the room and instructed participants to use hand signals to indicate if the therapist should raise or lower its volume during the session. The Nicolet station records synchronous video, and we marked the start/end of each condition post session at the EEG lab where sessions took place on the Nicolet recording software before export to edf+, which was then imported into the analysis software.

### EEG Signal Processing

We imported the EEG recordings, including reference channels and artifact recordings, into NeuroGuide analysis software (version 2.7.3) (Thatcher et al., 2011). The EEG traces were re-referenced to averaged linked ears using a montage set including both mastoid channels recorded with the EEG. The NeuroGuide software downsamples the EEG data to 128 Hz, and baselines and filters (<1 Hz and >55 Hz) the raw data with a 5th Order Butterworth filter. Using NeuroGuide’s artifact toolbox which calculates split-half and test–retest reliability measures for selected EEG data, as well as semi-automatic detection of eye movement, we selected reliable, artifact-free EEG traces for each condition from each participant’s data. We also visually inspected the EEG, ECG, and EOG recordings to manually exclude segments of data affected by eye movements, blinks, or muscle activity. NeuroGuide baselines and filters the artifact-free, spliced EEG selections a second time (at < 1 Hz and >55 Hz). Then NeuroGuide performs a Power Spectral Analysis (PSA) for the artifact-free EEG selections with a Fast Fourier Transform (FFT) and divides EEG selections for the FFT into 2-s epochs. This results in a frequency range of 0.5 to 55 Hz at a resolution of 0.5 Hz using a cosine taper window. NeuroGuide then divides the EEG data from each channel into predetermined frequency bands.^1^

### Statistical Comparisons

Table 2 presents the analyses we conducted on each set of EEG data using NeuroGuide’s statistical toolbox. Of the three cases, only one participant (Darryl^2^) reported experiencing pain at the time of the EEG study. Furthermore, one case (Will) had limited clean data available for analysis due to technical problems, therefore preventing analysis of data related to Propositions 1–3; however, we were able to extract reliable EEG traces from Will’s data for the fourth and final analysis, which enabled us to conduct a group paired *t*-test comparing all three participants’ personalized healing music condition to the preferred music condition (Proposition 4) with the Alpha level for all paired *t*-tests set at 0.05.

**TABLE 2:** Quantitative EEG analyses.

**Participant**	**Conditions**	**Analysis**
Darryl	Personalized pain music – other pain music	Individual paired *t*-test
	Personalized healing music – other healing music	Individual paired *t*-test
	Post personalized entrainment music rest – baseline rest	Individual paired *t*-test
	Personalized healing music – preferred music	Group paired *t-*test
Will	Personalized healing music – preferred music	Group paired *t-*test
Carolyn	Personalized healing music – preferred music	Group paired *t*-test

For the comparisons within the different entrainment conditions and the pre–post entrainment rest conditions (Propositions 1–3), we conducted paired *t*-tests on the absolute power of the usable EEG traces from each set of Darryl’s and Carolyn’s data. Based on these EEG data, we conducted low-resolution brain electromagnetic tomography (LORETA) to localize the EEG data to a three-dimensional brain model. Given Darryl’s reported change in pain response during the music intervention, we focused this report on Darryl’s data regarding propositions 1–3 as a case study.

Given that EEG frequency bands reflect different functions and behave statistically independently (Kubicki et al., 1979), Low Resolution Electromagnetic Tomography (LORETA) (Pascual-Marqui et al., 1994, 1999) can estimate the three-dimensional intracerebral current density distribution for the frequency bins as set in Neuroguide software. These distributions were used to estimate localizations of activity within a 3-D brain model, based on the Brodmann Areas (BA) that are in the core or nearest proximity of the LORETA solution space (2394 voxels with a spatial resolution of 7 mm) to which the LORETA algorithm projects the EEG source localization. The localization is restricted to cortical gray matter and hippocampi referring to the digitized Talairach and probability atlases of the Brain Imaging Center, Montreal Neurologic Institute (MNI305). BAs are associated with a distinct cytoarchitecture of neuroanatomical pathways, and through extensive empirical study, are correlated closely with particular neuropsychological functions as described in Kolb and Whishaw (2009). The LORETA version implemented within the Neuroguide software utilized in the current study utilizes a three-shell spherical head model registered to the Talairach brain atlas (Talairach and Tournoux, 1988) and EEG electrode coordinates derived from cross-registrations between spherical and realistic head geometry (Towle et al., 1993).

When conducting analyses in the frequency domain, LORETA values at each voxel represent the power (i.e., squared magnitude) of the computed intracerebral current density (units of amperes per square meter). To analyze raw source current values, Neuroguide uses Non-Transformed Raw Values or Square Root Transformed Raw Values. The non-transformed raw values are the squared source current vectors (i.e., square root of the sum of squares of x, y, and z) with units of (amperes^2^/meter^2^)^2^ and the units for the square root transform of the squared source current vectors are amperes/meter^2^. To check against incorrect use of input electric potential power (Pascual-Marqui, 2002), we ran all LORETA analyses using both Non-Transformed Raw Values and Square Root Transformed Raw Values and compared output for differences; we found no differences in LORETA output in our comparisons. For more detailed discussion on the calculation of these statistics, we refer you to Appendix H of the *Neuroguide Help Manual* (Thatcher, 2018).

## Results

The EEG results reported here are twofold:

(I) Single case results (Darryl) regarding propositions 1–3: regarding his active listening to (1) The two entrainment music segments [(a) pain and (b) healing music] against control music (both entrainment music segments from another participant) and (2) pre/post entrainment resting-state comparison.
(II) Group case results (Darryl, Will, and Carolyn) regarding proposition 4: on the difference of resting states after entrainment healing music compared to preferred music.

### Darryl: Case Results

#### Darryl’s Clinical Background

Darryl was under treatment for a hematologic tumor at the time of the study. According to the assessment from his referring physician, his pain was neuropathic in nature and had been ongoing for more than a year. The pain was in his lower spine and hip, and he reported this resulted from bone damage. When he enrolled in the study, he was taking between 30 and 100 mg of narcotic medication daily to manage his pain, which he reported would come and go, and would build in intensity “like a toothache – after a while it starts throbbing.”

In his interview after completing Phase 1 of the study, Darryl shared that he found both the preferred music and the entrainment music conditions helpful for relieving his pain. During the preferred music, he stated that he attempted to focus on the music along with images and memories of his family to distract himself from feeling pain. During the entrainment condition, he sought to focus on the different instruments and the therapist’s singing. He said that this was effective in “taking my mind off being hurt.” He stated there was not much difference in pain relief between the two conditions, though he also similarly approached both conditions by concentrating on the music moment-by-moment. However, several times he reflected on the immediacy and physical activity of the entrainment condition, saying, “the live music is right there, you can see the person performing, concentrating and stuff.” However, though the music helped him feel some relief from the pain, as soon as either music intervention stopped during the sessions, the pain returned. For a detailed description of the duration and characteristics of each of the music segments, see Appendix A.

#### Darryl’s Pre–Post EEG Pain Ratings and Reported Responses to Music for Pain

At the time of the EEG study, Darryl described his pain as having a throbbing sensation in his lower back; his pain ratings were unchanged from pre to post EEG session (4/10 to 4/10). However, he stated after the EEG study that both entrainment conditions (personalized and control, see Figure 2) met his pain/healing needs and that the preferred music was also helpful. He felt that his own healing music was the most helpful out of all the conditions, as he could recall the lyrics of the familiar hymn tune used for this segment (the lyrics were not sung in this case, though the therapist vocalized the melody), and he enjoyed the way the therapist performed it. He also stated that “when I listen to the music, I don’t even acknowledge or feel [the pain is] there… music has a way of taking away pain – to me, it takes the focus off the pain.”

#### Darryl’s EEG Results

##### Proposition 1: comparing Darryl’s personalized pain music with control pain music (Entrainment)

This proposition stated that when participants listened to entrainment music depicting the participant’s “pain” experience versus the music depicting a non-participant’s “pain” experience as control, we expected differences in theta, beta, and/or gamma range.

**FIGURE 3:**
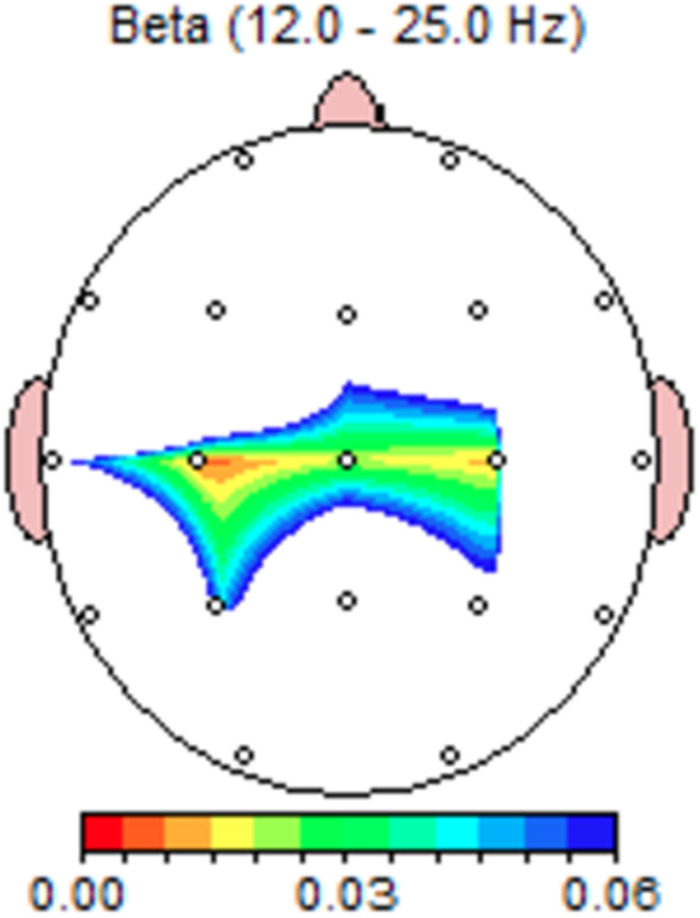
Darryl’s paired *t*-test significance map at beta frequency range for Personalized-Control Pain music conditions; Personalized Pain music induced changes in sensory-motor regions (Proposition 1). Colors indicate significance levels.

Paired *t*-tests showed no significant changes in theta frequencies. Significant differences occurred in the beta range from 12 to 18 Hz, with power increases for the personalized pain music along central electrodes C3, Cz, C4, representing sensorimotor areas (see Figure 3). Table 3 presents these significant changes and results of the LORETA analysis at Beta frequency range (12–18 Hz), including regions with highest *t*-values and significance level at left middle temporal gyrus, right inferior frontal gyrus/inferior temporal gyrus, superior temporal gyrus, and middle occipital gyrus. There were no significant differences at the gamma range for this proposition.

**TABLE 3:** LORETA results for Darryl: Propositions 1–3.

**Frequency Bin**	**Electrodes**	**Direction of Change**	**Brodmann Area (BA) of corresponding peak *t*-value**	***p-*value**
**P1: Own Pain vs. Control Pain music (Entrainment)**				
12–18 Hz	C3, Cz, C4	Increase	Left MTG (BA21)	0.001
			Right IFG (BA44)	0.008
			Right MFG (BA6)	0.001
			Right ITG (BA21)	0.017
			Right STG (BA22)	0.010
			Right middle occipital gyrus (BA19)	0.001
			Left post-central gyrus (BA 1, 2, 3)	<0.001
			Right post-central gyrus (BA 1, 2, 3)	<0.001
**P2: Own Healing vs. Control Healing music (Entrainment)**				
8–10 Hz	T6, P4, Fp2	Increase	Right SMG (BA40)	< 0.001
			Right Precuneus (BA19)	0.001
13–15 Hz	F4, Fz, Cz, Pz	Increase	Anterior cingulate (BA33)	0.001
			Cingulate gyrus (BA24)	0.001
			Right ITG (BA20)	0.001
			Left SFG (BA11)	<0.001
**P3: Post entrainment condition rest vs. post baseline rest**				
15–17 Hz	Cz, P4, T6, Fz, F4, F8, T4	Increase	Right Parietal cortex (BA40),	<0.001
			Right Precuneus (BA7)	<0.001
			Right ITG (BA20)	<0.001
28–40 Hz	P3, O1, F7, T3	Decrease	Broca’s area (BA20)	<0.001
			ITG (BA44)	<0.001
			Left primary auditory cortex (BA42)	<0.001
			Left postcentral gyrus (BA43)	<0.001
			Left insula (BA13)	<0.001
			Left primary motor cortex (BA4)	<0.001
28–40 Hz	F4, C4, P4, O2, T4, T6	Increase	Right insula (BA13)	<0.001
			Right ITG (BA20)	<0.001
			Right MTG (BA21)	<0.001
			Right parietal cortex (BA40)	<0.001

*MTG, medial temporal gyrus; IFG, inferior frontal gyrus; STG, superior temporal gyrus; SMG, supramarginal gyrus; SFG, superior frontal gyrus; ITG, inferior temporal gyrus; MTG, medial temporal gyrus.*

##### Proposition 2: comparing Darryl’s personalized healing music to control healing music (Entrainment)

This proposition stated that when participants listened to entrainment music depicting the participant’s “healing” experience versus the music depicting another participant’s “healing” experience as a control, we expected power changes in theta, beta, and/or gamma range.

**FIGURE 4:**
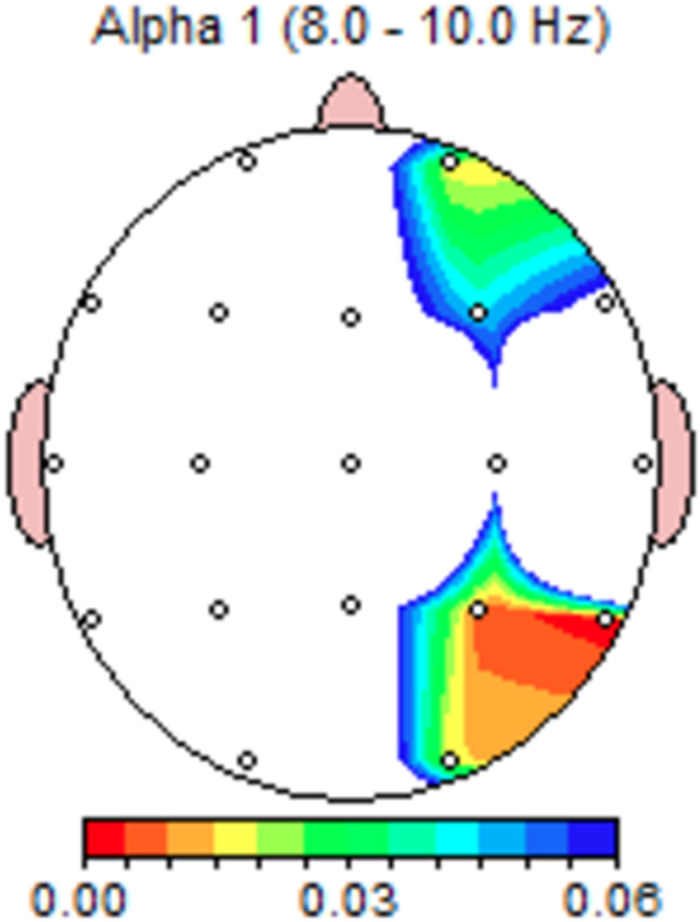
Darryl’s paired *t*-test significance map at alpha-1 frequency range for personalized-control healing music conditions (Proposition 2). Colors indicate significance in right frontal and parietotemporal areas.

There were no significant changes at theta (4–8 Hz), however, LORETA analysis at 8–10 Hz found the highest *t*-values indicating activity change in inferior parietal lobule and precuneus (Table 3 shows significant electrode sites and Figure 4 illustrates paired *t*-test significance). These changes are notable given Stern et al. (2006) defined high theta range at 6–9 Hz, and in this range found overactivation in chronic pain patients.

Personalized healing music elicited significantly increased power in the low beta range at F4, Fz, Cz, and Pz. LORETA analysis at 13–15 Hz indicated highest *t*-values for increased activity in the anterior cingulate, cingulate gyrus, ITG, and left SFG (Table 3). There were no significant changes at gamma power in this comparison.

##### Proposition 3: Comparing Darryl’s resting state after entrainment music to baseline rest

This proposition stated that we expected differences in absolute power at theta, beta, and/or gamma frequencies when comparing the resting period after the entrainment condition (including both pain and healing music) to the first period of eyes-closed, resting condition in silence.

Paired *t*-tests showed no significant differences in absolute power at the delta or theta ranges, however, there were significant changes at beta frequencies at midline sites. LORETA analyses (Table 3) showed increased beta power in the parietal cortex, precuneus, and ITG. At the high beta and lower gamma range (28–40 Hz), left hemisphere activity decreased. LORETA imaging indicated these changes occurred in Broca’s area, ITG, primary auditory cortex, primary gustatory cortex, insula, and primary motor cortex.

**FIGURE 5:**
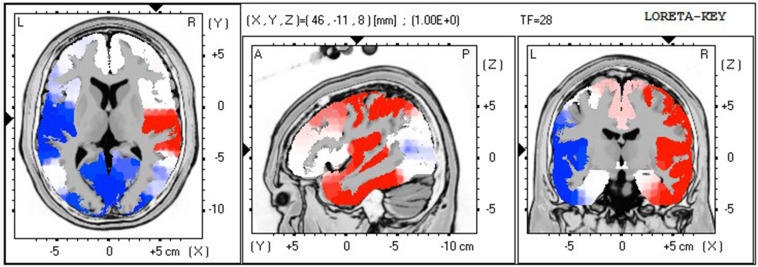
Darryl’s *p*-value LORETA image at 28 Hz, comparing post entrainment music rest period with baseline rest (Proposition 3). This example of activity changes in the high frequency ranges (observed in 22–40 Hz) shows the overall change of the right hemisphere (red: increase of activity in Insula, Temporal, and Postcentral Gyrus) after listening to entrainment music.

Simultaneously, gamma activity (between 34 and 39 Hz) significantly increased in the right hemisphere, involving the insula, ITG, MTG, and parietal cortex. Additionally, central sites at the gamma frequency showed significant decreases in the SFG. Figure 5 shows the corresponding *p*-value LORETA image at 28 Hz.

#### Integration of Interview and EEG Results for Propositions 1–3

Interviews conducted with this participant after undergoing both the Entrainment and Preferred music sessions in Phase 1 indicated that the participant experienced both intentional moment-by-moment attention on the sounds/music rather than on pain which led to the participant’s decreased awareness of his pain. These experiences could relate to the EEG data in these ways:

Proposition 1 (personalized pain vs. control pain music): Darryl had remarked during Phase 1 that he found himself noticing the therapist’s motions as she played the entrainment music, and this brought the music “right there” to the location of his pain. When comparing neuronal responses obtained while Darryl listened to music composed for his own pain compared to music composed for another person’s pain, his sensorimotor regions were more active, as were auditory and language processing regions. In addition, the IFG has been implicated in processing both melodic and rhythmic musical improvisation (Berkowitz and Ansari, 2008), as well as visual-motor activity related to reading music (Sergent et al., 1992; Bengtsson and Ullén, 2006); this latter result might imply that Darryl actively responded to the music he created with the music therapist in a manner consistent with musicians improvising music, whereas he did not respond in this way to the control music he did not create with the therapist.

Proposition 2 (personalized healing vs. control healing music): Given Darryl’s report that the healing music alleviated his pain, it seems that the EEG results here more likely point to the inhibitory function of alpha frequencies in the precuneus and the inferior parietal gyrus (whose functions include somatosensory integration, pain, and body image; Cavanna and Trimble, 2006; Shomstein, 2012) rather than the overactivation at 6–9 Hz in somatosensory regions Stern et al. (2006) observed in patients with chronic pain at rest. Increased beta power in the anterior cingulate, cingulate gyrus, ITG, and left SFG may relate to active attention and concentration on functions in these regions. Other imaging methods have shown that the anterior cingulate is associated with motor imagery (Munzert et al., 2008), the ITG with visual-motor integration (Indovina et al., 2016), and the SFG with introspection (Goldberg et al., 2006) and reward anticipation (Ernst et al., 2004). These observations align with Darryl’s description of his moment-by-moment focus on the active music-making during the entrainment condition, including his awareness of the therapist’s movements and concentration while playing. Involvement of reward processing might relate to Darryl’s choice of the melody of a familiar, comforting hymn for the healing portion of the entrainment condition (see music description in Appendix A).

Proposition 3 (resting state after entrainment vs. baseline resting state): Darryl reported after the entrainment session in Phase 1 that “I could tell it was working because I was more relaxed, concentrating on the music.” In the rest period immediately after his personalized entrainment music, LORETA indicated increased somatosensory and visual processing as well as increased right hemisphere activity for auditory and visual processing. The gamma power increase may reflect cross-modal processing of auditory phenomena, working memory, and interoceptive awareness in the right hemisphere, whereas corresponding functions, as well as memory, music perception, and primary motor functions, decreased in the left hemisphere. This lateralization is clear in Figure 5 and seems to be consistent with Darryl’s report of integrating his physical awareness with his experience of the music and reflection on his relaxed state after the conclusion of the entrainment condition.

Thus, regarding Darryl’s experience of the entrainment music, the data for each of these comparisons focusing on both pain and healing music showed involvement of visual-spatial and motor imagery areas, as well as areas related to improvising music during the pain music. These responses could reflect Darryl’s recollection of creating the entrainment music with the therapist, as well as his somatosensory experiences corresponding to his moment-by-moment focus on the music as he listened to it.

### Group Results

This section begins with clinical background for the remaining two participants (Will and Carolyn), followed by group analysis of all three participants’ data on Proposition 4. The music therapist compiled the music conditions for both Will and Carolyn in the same manner as for Darryl, following the block design (Figure 2). Appendix A contains details of the duration and musical characteristics of their music conditions.

#### Will’s Clinical Background

Will had been experiencing pain for less than a year when he enrolled in the study. The pain was in his head and jaw and caused by a solid tumor. He had difficulty opening his jaw due to the pain. He reported upon enrollment in the study that the pain would come and go and would often resolve after taking PRN medication. In the interview after participating in both the preferred music and the entrainment music conditions, Will stated that he preferred the commercial music because of his ability to relax and “meditate” on the music experience. In contrast, the entrainment experience required him to interact with the therapist throughout, which he felt made it difficult for him to fully relax and focus on the music. He stated he preferred listening to soft music, relaxing to the point where he would fall asleep, and the pain would be gone.

#### Will’s Pre–Post EEG Pain Ratings and Reported Responses to Music for Pain

Will rated his pain as a 0/10 both before and after the EEG study. After the study he explained that his entrainment music “somewhat” matched his pain/healing needs; he said as he was not in much pain he could not really state the relationship between the music and his pain, though he “liked” the music, and it “sounded nice.”

#### Carolyn’s Clinical Background

Carolyn had been coping for more than a year with both somatic and neuropathic pain in her upper legs and knees because of a solid tumor. At the time of enrollment in the study, she was not undergoing active therapy for the tumor. She was taking less than 30 mg of narcotic pain medication per day and was also taking anticonvulsant adjuvant medication for pain. At the time of enrollment in the study, she reported that her pain was constant, usually rating around a 3–4 on a 1–10 scale of intensity.

In the interview after participating in Phase 1, Carolyn stated that she found the entrainment condition “entertaining” and that she preferred the therapist’s piano and guitar playing for easing her pain rather than the sound of live drums. She felt she was more relaxed during the preferred music condition, but that she felt more “alive” during the entrainment condition: “It brought me back to life. The recorded music settled me down a little.” She felt both conditions served as a distraction from her pain; while her pain did not disappear completely, “I felt a difference.” She also referred to sound images that came to mind during both music conditions that helped distract her from pain, such as the sounds of ocean waves breaking on a beach.

#### Carolyn’s Pre–Post EEG Pain Ratings and Reported Responses to Music for Pain

At the start of the EEG study, Carolyn stated that she had pain in her left knee that she rated at a 3/10; at the end of the study, the pain was slightly worse, between a 3–4. In the post-EEG interview, Carolyn stated that her own healing music caused her more pain, which she described as “banging in my leg.” Thus, it did not match her healing needs, but her own pain music matched her experience of her pain. She felt the preferred music was “a little bit relaxing.” The control entrainment music “didn’t really help [the] pain any… it was not my music.”

#### Proposition 4: EEG Group Analysis Comparing Personalized Healing Music to Preferred Music Condition

In this final analysis, our proposition stated that, for the group of three participants, we expected that the personalized healing music would lead to changes in delta, theta, beta, and gamma power compared to the preferred music. For this proposition, we pooled the EEG data for all three participants to conduct a group paired *t*-test on the absolute power difference between participants’ listening to their personalized healing music from the entrainment condition minus their preferred music condition. Table 4 lists these significant differences according to each frequency bin, electrode site, Brodmann area, and significance level.

**TABLE 4:** Group comparison of localized differences between personalized healing entrainment and preferred music conditions according to 1 Hz frequency bins, electrode, and source estimation.

**Frequency Bin**	**Electrodes**	**Direction of change**	**Brodmann Area (BA) of corresponding peak *t*-value**	***p-*value**
2 Hz	F7	Decrease	Left Uncus (BA 36)	<0.001
			Left insula	<0.001
			Left STG	<0.001
3 Hz	T3	Decrease	Right MTG and Right ITG (BA 20)	<0.001
4 Hz	Cz, C4	Decrease	Right parahippocampal gyrus (BA35)	<0.001
			Right SFG (BA10)	<0.001
			Right MFG (BA 11)	<0.001
6 Hz	F3, C3, P3 Cz	Decrease	Left MFG (BA 9, 10)	0.001
15 Hz	T6	Increase	Right precentral gyrus (BA6)	0.010
16 Hz	Fp2	Increase	Right ITG (BA20)	0.001
17 Hz	Fp2, F8	Increase	Right inferior parietal lobule (BA 40)	<0.001
			Right post central gyrus (BA2)	<0.001
			Right Insula (BA13)	<0.001
18 Hz	C4	Increase	Right post central gyrus (BA3)	<0.001
25 Hz	O1	Increase	Right Parahippocampal gyrus (BA28)	<0.001
			Right Posterior cingulate (BA30)	<0.001
28 Hz	Cz	Increase	Parahippocampal gyrus (BA 27)	<0.001
			Right hippocampus (no BA)	<0.001
29 Hz	C3	Increase	Right parahippocampal gyrus – fusiform gyrus (BA 36)	0.001
30 Hz	F4	Increase	Parahippocampal gyrus (BA 35)	0.002

*n = 3; Linked ear reference; Absolute power paired *t*-test changes in 1 Hz frequency bins and according to LORETA source estimations (regions representing peak *t*- and *p*-values; compare Figure 5). MTG, medial temporal gyrus; IFG, inferior frontal gyrus; STG, superior temporal gyrus; SMG, supramarginal gyrus; SFG, superior frontal gyrus; ITG, inferior temporal gyrus; MTG, medial temporal gyrus.*

Delta frequency (1–4 Hz) changes were significant at F7, T3, Cz, and C4; LORETA analysis indicated significant decreases in limbic structures as well as decreases in left STG, right SFG and MTG, and right MFG.

Significant differences occurred at the theta frequency at F3, C3, P3, Cz (6 Hz), and C3 and F3 (8 Hz). LORETA analysis identified decreased power in the right MFG and increased power in the right STG.

**FIGURE 6:**
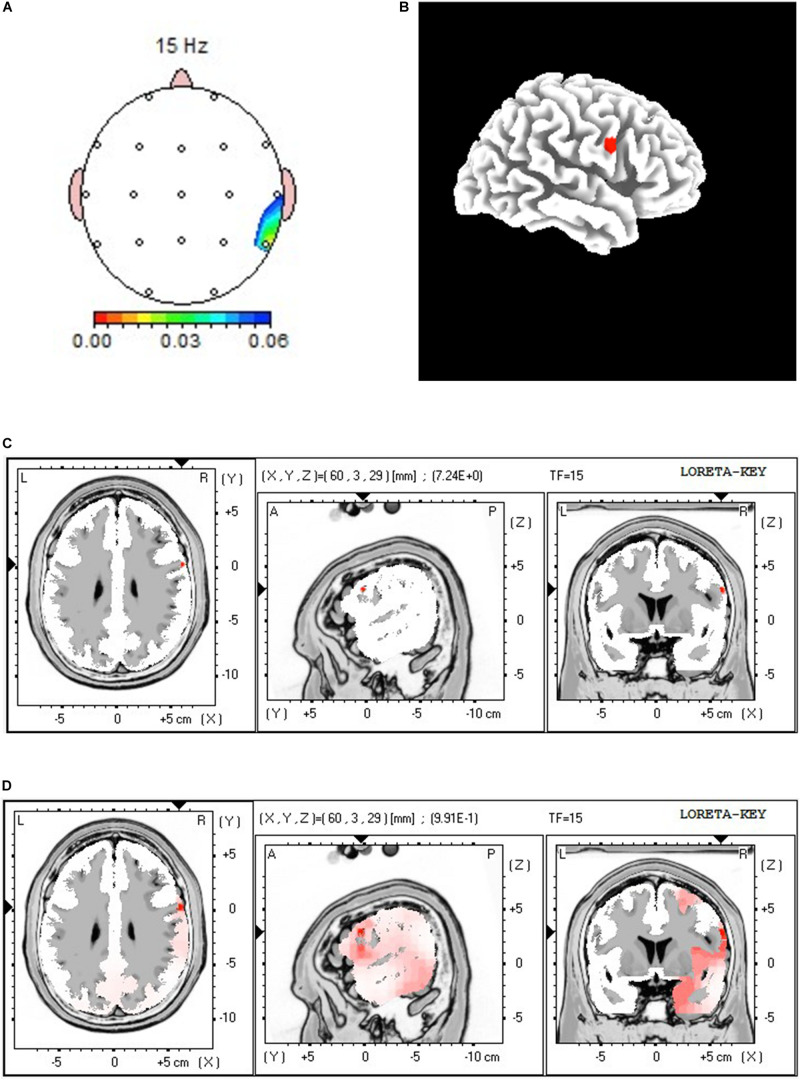
Results of group comparison of Personalized Healing and Preferred music conditions (Proposition 4). **(A)** Paired *t*-test 15 Hz significance mapping, **(B)** 3-Dimensional Map of LORETA *t*-value at 15 Hz, **(C)** *t*-value LORETA image at 15 Hz, **(D)** *p*-value LORETA image at 15 Hz. Colored areas on image **(A)** indicate significant changes at T4 and T6; red areas on LORETA images [exact *t*-value location in **(C)**; probability spread of increase in **(D)**] indicate increased power and lateralization of activity during the Personalized Healing condition.

*T*-tests showed several significant changes at the beta frequency. At the low beta range, differences occurred at C4 (12 and 18 Hz), T6 (15 Hz), Fp2 (16 and 17 Hz), and LORETA analysis showed increased power in right hemisphere somatosensory areas and primary motor cortex. LORETA analysis also indicated significant increased power at 12 Hz in the right cuneus, and right superior and middle occipital gyrus. Figure 6 shows the paired *t*-test, 3-dimensional map of LORETA *t-*value, and *t-*value LORETA image at 15 Hz for this comparison, indicating increased beta in the somatosensory cortex in the right hemisphere. At the upper beta range, significant changes occurred at O1 (25 Hz) and Cz (28 Hz), and C3 (29 Hz). In connection with these changes, LORETA analysis identified limbic system activity increased in the parahippocampal gyrus, hippocampus, posterior cingulate, and fusiform gyrus. There were no significant differences beyond 29 Hz, therefore there were no changes in gamma power for this proposition.

#### Integration of Interviews and EEG Results for Proposition 4

Despite different subjective reports, the group analysis revealed a specific brain response in the right hemisphere somatosensory areas to their personalized healing and preferred music conditions, as evidenced between both conditions in the theta and low beta range (Table 4 and Figure 6). Furthermore, the participant reports suggested involvement of visual, sensorimotor, and motor processing.

Subjective reports from all three participants revealed mixed responses to their personalized healing music compared to the preferred music condition at the time of the EEG studies. As discussed in the first case study, Darryl felt that listening to his personalized healing (entrainment) music and his preferred music were both very helpful for his pain, whereas Will experienced no pain during the EEG session and had a neutral stance on the effect of the music on his pain, and Carolyn had a negative reaction to both the music conditions with slightly worsening pain as a result.

The significant decreases in the delta range involved parahippocampal, frontal, and temporal structures, implicated in functions such as working memory, memory encoding, experiencing/processing emotion, and novelty discrimination. These are compatible with the descriptions of the participants’ engagement with the entrainment conditions, for example, Carolyn’s description of this music as being “entertaining” and Will’s awareness of the amount of attention on the therapist the entrainment music required. These phenomena are also consistent with the decreased theta power in the MFG, which has been associated, for example, with cognitive branching (BA10) and working memory, memory retrieval, encoding, selective attention to sounds, executive function, behavioral inhibition, processing negative emotional stimuli, and calculation (BA 9).

The observed high theta/low alpha increased power may involve auditory processing, specifically familiar voices (right pole) and aversive auditory stimulation (see Zald and Pardo, 2002) and familiar voices (Nakamura et al., 2001). Given the prominent involvement of the music therapist’s voice in Darryl’s healing music and the overall harmonic nature of all three segments of healing music, it seems more likely these phenomena were actively functioning at theta rather than inhibited by alpha frequencies.

The increased low- and high-beta power changes prove highly interesting. Together they implicate increased somatosensory, motor, and visual processing, along with increased hippocampal and parahippocampal activity potentially involving memory encoding and emotion formation, in addition to increased power in the posterior cingulate, which has been linked to processing pain and episodic memory (Nielsen et al., 2005). When considered with the decreased delta power observations, the location and direction of these beta changes correspond to the participants’ recall of the music-making with the therapist, and their awareness of their body responses to the entrainment music. For example, as seen in Darryl’s responses to the entrainment conditions, these responses may reflect the participants’ recollection of the therapist’s movements to play the instruments for the participants’ personalized sessions.

## Discussion

Sarnthein et al.’s (2006) and Stern et al.’s (2006) studies examining neuronal oscillations in patients with chronic neurogenic pain utilized continuous EEG to discover an overactivation of high theta (6–9 Hz) and low beta (12–16 Hz) power in central regions. These two studies have interesting implications for Darryl’s case but in two different ways. First, Darryl’s paired *t*-test comparing his own versus control *pain* music from the entrainment condition showed increased somatosensory activity at the low beta frequency range. In this case, the music created by this patient and improvised for him, which was intended to represent his pain, induced neuronal activity consistent with that seen in patients with chronic neurogenic pain in Sarnthein et al. (2006) and Stern et al. (2006). Second, in comparing his own healing music to the control healing music, Darryl reported that his music alleviated his pain (neuropathic in nature), and concurrently activity in the 8–10 Hz range (classified as alpha by Neuroguide) increased in the right SMG and precuneus. In this instance, given Darryl’s report of alleviated pain, these neuronal responses may reflect the inhibitory function of alpha rather than overactivation of theta seen in Stern et al. (2006).

Meanwhile, the group paired *t*-test comparing *personalized healing* music compared to *preferred* music showed significant decreased power at the theta frequency range at Cz, C3, and C4; thus, there was no overactivation at this frequency observed among the group. Furthermore, the decreased delta observed in this comparison does not seem to correspond to changes seen in Hauck et al. (2013) which occurred in the centroparietal and somatosensory cortex during the preferred music condition. Thus, these decreases in delta and theta indicate a strong response at these frequencies to the participants’ own entrainment-healing music compared to the preferred music condition.

Related research demonstrates increased alpha power evoked from meditation intervention corresponding with decreased reports of pain. Jensen et al. (2013) found that separate meditation and hypnosis provided to patients with chronic neuropathic and mixed (neuropathic and nocioceptive) pain led to both decreased pain ratings and increased alpha in post-treatment resting conditions. Specific changes included increased central–left parietal activity after meditation and widespread alpha after hypnosis. Though not the focus of our propositions, we observed consistent findings in Darryl, in the comparison of Own healing vs. control healing (P2), showing increased alpha at supramarginal gyrus and precuneus (Table 5). Though alpha also increased in the group comparison of personalized healing vs. preferred music (P4), these changes occurred in postcentral gyrus, left posterior cingulate, and STG (Table 6); In contrast with our findings, Jensen et al. (2013) found increased theta after meditation (left frontal and posterior regions) and no changes in theta after hypnosis. Whereas our findings were consistent with Jensen et al. (2013) regarding a single participant’s response to his personalized healing music, the similarities end there. The unique properties of the music experiences in our study may have been a factor in the location of increased alpha and the decreased theta activity seen in the group comparisons.

**TABLE 5:** LORETA results for Darryl; Proposition 2 (own healing – control healing), alpha range.

**Frequency Bin**	**Electrodes**	**Direction of Change**	**Brodmann Area (BA) of corresponding peak *t*-value**	***p-*value**
8–10 Hz	T6, P4, Fp2	Increase	Right SMG (BA40)	<0.001
			Right precuneus (BA19)	0.001

*Linked ear reference; Absolute power paired *t*-test changes in 1 Hz frequency bins and according to LORETA source estimations (regions representing peak *t*- and *p*-values; compare Figure 5). SMG, supramarginal gyrus.*

**TABLE 6:** Group comparison of localized differences between personalized healing entrainment and preferred music conditions at alpha range according to 1 Hz frequency bins, electrode and source estimation.

**Frequency Bin**	**Electrodes**	**Direction of change**	**Brodmann Area (BA) of corresponding peak *t*-value**	***p-*value**
8 Hz	C3, F3	Increase	Right STG (BA 38)	0.001
10 Hz	Fp1, Fz	Increase	Right postcentral gyrus (BA43)	0.002
12 Hz	C4	Increase	Right cuneus	<0.001
			Superior and middle occipital gyrus (BA19)	<0.001
			Left posterior cingulate (BA31)	0.001

*n = 3; Linked ear reference. STG, superior temporal gyrus.*

Hauck et al. (2013) found that entrainment music appeared to induce gamma band activity in the somatosensory (SI) cortex, whereas commercially recorded music correlated with delta activity in the midcingulate cortex and contralateral insula. Specifically, the authors found that the pain portion of the entrainment music correlated with increased gamma band activity in the SI cortex; this same region and frequency also showed a strong relationship to the laser-induced pain stimuli alone. The same subjects also rated the positive/negative valence of the pain as well as the intensity of the pain, and the correlation of these ratings with the music conditions found that both the pain and healing music from the entrainment condition were significantly associated with unpleasantness and intensity ratings of the pain as well as gamma-band activity in the somatosensory cortex. In our study, Darryl’s post-entrainment LORETA imaging did show significant changes at the gamma range at the right insula, and temporal and postcentral gyrus. At the delta range, the present study only revealed significant increases in delta power in frontal regions in the comparison of personalized versus control pain music (entrainment music) for Darryl.

**FIGURE 7:**
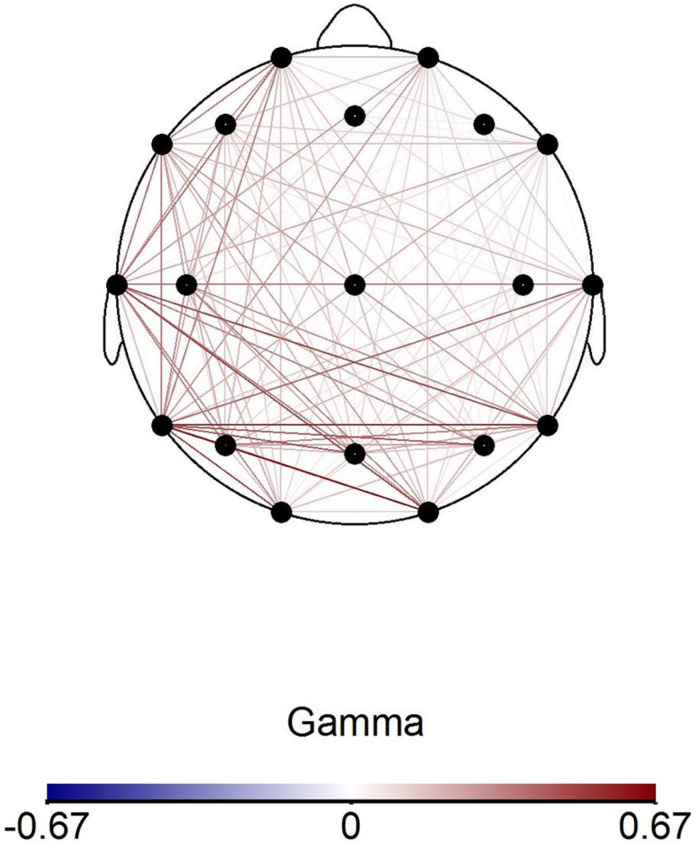
Darryl’s coherence map at gamma frequency range comparing average absolute power values during Own Pain (entrainment)-Control Pain (entrainment) conditions. Connected electrode pairs in red indicate stronger coherence during Own Pain music condition compared to Control Pain condition.

Though our results only show involvement of the SI cortex at the beta frequency, the involvement of Darryl’s insula at the gamma range (Proposition 3) may reflect Hauck et al.’s (2013) conclusions, which argued that participants’ improvised pain and healing music can modulate sensory perception at an early stage of processing. Hauck et al. (2013) noted the binding and cross-cortical information-transfer properties of gamma coherence (Fries, 2005), and that gamma coherence is enhanced during attentional selection of sensory information (Herrmann et al., 2004). Furthermore, cortical synchrony (or Imaginary Coherence, as calculated in Hauck et al., 2007) at the gamma range may indicate re-routing pain signals across cortical layers toward limbic structures, which are involved in emotional processing, monitoring, and descending control of pain (Lorenz and Garcia-Larrea, 2003). Therefore, we undertook secondary *post hoc* analysis^3^ of Darryl’s gamma coherence comparing neuronal responses to his own pain music from the entrainment condition to the control pain music and found a large number of widespread, cross-hemispheric networks during his own pain music (Figure 7).

In addition to research that supports a top–down influence through gamma activity in response to pain (Gross et al., 2007; Hauck et al., 2013), other studies have shown that when subjects perceive pain as controllable, the brain recruits top–down mechanisms of pain modulation (Moll et al., 2002; Wiech et al., 2008). However, gamma responses are most prominent in studies where pain was induced, and authors discuss gamma binding in the experience and modulation of the acute pain stimulus (Pinheiro et al., 2016; Ploner et al., 2017); in contrast to these studies, our patients had chronic pain. Nevertheless, the LORETA images in Figure 5 and the gamma coherence increase shown in Figure 7 may depict this top–down process in Darryl during the pain portion of his own entrainment music. The top–down processing theory fits with the rationale for Entrainment as an intervention, in that the music therapist seeks to help clients perceive their pain more objectively and as a more manageable phenomenon (Dileo and Bradt, 1999).

Our results are unique compared to most of these related studies in that we observed more right-hemispheric lateralization in our participants, including in the high frequency ranges of the resting EEG changes from pre- to post entrainment music in Darryl (see Figure 5). Such lateralization has been consistently related to music processing in electrophysiological studies of the auditory domain (Tervaniemi and Hugdahl, 2003). Interestingly the *group* differences of the music conditions showed predominant right hemisphere activity for participants’ personalized healing music (Figure 6), which may indicate a different kind of involvement and processing of entrainment music compared to preferred music. Further research could determine whether such lateralization is a common phenomenon for patients with chronic pain during entrainment music, and how this lateralization affects pain perception and modulation.

In addition, the involvement of visual-spatial, motor, and language/music improvisation processing in Darryl’s responses to his own entrainment music may reflect the active recollection of creating this music with the music therapist. In interviews following both phases of the project, he commented on his recollection of the therapist performing the music; after the first phase, he specifically referred to the visual modality, stating, “you can see the person performing, concentrating and stuff.” These reports seem to support the involvement of the processing observed in our data. It is possible that these results reflect some elements of the interpersonal process of the intervention. In listening to recordings of music for EEG acquisition, the participants might have recalled the live music intervention created with their input and performed by a music therapist with whom they had discussed, in detail, their pain experience as well as a sonic representation of pain relief. This potential recollection of the interpersonal processes provides an added relational dimension of active engagement in the music intervention, whose implications in pain processing require further investigation. This is in contrast to the participants’ reports of their internal focus during the preferred music condition, which involved memories, images, and concentrating on the music as a form of meditation. Thus, though Hauck et al. (2013) characterized the preferred condition as “music as distraction” and the Entrainment intervention as “music as active coping,” the qualitative data suggest the possibility that these cases could be examples of “music as intrasubjective coping” (Preferred Music/Music Medicine) and “music as intersubjective coping (Entrainment/Music Therapy).

Preliminary findings regarding musical reward as a pain inhibitor due to activation of the NAc and midbrain nuclei have guided music therapists in developing pain interventions that also influence mood and cognitive responses to the pain (Chanda and Levitin, 2013; Bradt et al., 2016b). Darryl’s description of his experience during both music interventions appear to mirror this process, and LORETA localization of the involvement of the right insula at the gamma frequency in the comparison of post-entrainment rest vs. baseline rest may also reflect Darryl’s interoception (Craig, 2002). However, since our imaging lacked the capability of determining the involvement of the midbrain nuclei, this possible connection remains open to further investigation. Future studies should incorporate imaging such as PET or fMRI to validate these mechanisms and pinpoint their location.

## Limitations and Conclusion

This preliminary research examines only three cases, with mixed results in terms of pain management at the time of the EEG studies. For practical purposes, we did not control for the effects of the participants’ medications on the EEG. However, we are aware that opioid medications such as Tramadol have been reported to lead to changes in alpha and beta frequencies, with dose-dependent decreased power in high alpha (Thürauf et al., 1996), whereas Oxycodone has been shown to lead to decreased spectral indices and brain source activity in delta and theta frequency bands (Lelic et al., 2017). Thus, our results may have been influenced by the medications the participants were taking at the time, yet most pharmacological EEG studies (for example in depression) analyze responses during resting states (Saletu et al., 2010) in contrast to our comparison and analyses of responses to music interventions. On the other hand, these participants served as their own controls, and comparisons were made (at least for Darryl) within-subject, thus any difference between conditions should not be strongly influenced by pain medication. Therefore we do not expect any major confounding issues from the participants’ pain medication alone.

Regarding limitations to spatial resolution in our EEG analyses, we had access to a clinical EEG acquisition station and software platform certified for clinical diagnostics that included 21 channels. Whereas Michel and Brunet (2019) recommend a set-up of at least 32 EEG channels, they also state that “results do not necessarily mean that imperfect spatial sampling precludes source localization. Even with <32 electrodes, source localization allows to gain valuable insight about the underlying sources. “ (p. 8). The FDA-approved Neuroguide software embedded the LORETA version with 2394 voxels which allows cubicles of 7 mm to be separated, with which we used LORETA implementation for our explorations. Furthermore, the FDA has approved Neuroguide with LORETA for use with 21 channels because this setup is standard in clinical settings.

We also recognize the diminished ecological validity of using pre-recorded entrainment music with these patients. Whereas we would have preferred to provide live entrainment music and obtain rich qualitative data at the time of the EEG studies, the logistics of doing so in a working neurology clinic with participants being treated for chronic pain limited our ability to examine neuronal and subjective responses to a true clinical intervention. One consequence of this limitation was highlighted by Carolyn’s case, where her pain experience at the time of the EEG study did not match the music we provided. Yet the purpose of this initial investigation was to focus on positive pain responses to these music interventions, and future research should include comparisons with different or unexpected, negative responses. Despite these ecological limitations and the mixed effects of the music conditions on the participants’ pain ratings, both the individual and group results corroborated previous research on brain responses in individuals with chronic pain, and identified some unique responses that appear to relate to the nature of the entrainment condition. We also note that these case studies provide additional evidence to support the proposition of top-down versus bottom-up processing of pain modulation according to the functions of music as intersubjective coping (Music Therapy/entrainment music) versus music as intrasubjective coping (Preferred Music/Music Medicine), respectively. Accordingly, the results from the present study bear out Pinheiro et al.’s (2016) recommendation regarding the utility of qEEG to investigate neuronal responses to pain, and particularly for investigating responses to Entrainment and preferred music interventions for chronic pain relief. In addition to utilizing live entrainment music to ensure ecological validity, future research should investigate the degree to which these different music interventions utilize different neural networks, and how these networks are affected by variables such as pain and other medications, music and sound variables, and type of pain. To investigate the EEG of the intersubjective experience of interventions such as entrainment, researchers would need to involve the therapist’s brain activity in a hyperscanning investigation (Hunt, 2015; Fachner et al., 2019). Such information is essential to understanding the mechanism of these greatly needed interventions to add effective, beneficial, and low-risk pain relief options to patients.

## Data Availability Statement

The raw data supporting the conclusions of this article will be made available by the authors, without undue reservation.

## Ethics Statement

The studies involving human participants were reviewed and approved by Temple University IRB (Institutional Review Board). The patients/participants provided their written informed consent to participate in this study.

## Author Contributions

AH and JF drafted the manuscript. AH managed the project. AH, JF, and CM analyzed the EEG data. RR, RC-V, and CD designed the project and obtained the funding. RR, CM, and CD edited the manuscript. CR-K was the clinician and edited the clinical method and the music selections of the manuscript. RC-V recruited the participants. All authors contributed to the article and all living authors approved the submitted version.

## Conflict of Interest

RR is cofounder of CaRafe Drug Innovation, and CSO of Neumentum, both companies are directed to the discovery and development of non-opioid analgesics. The remaining authors declare that the research was conducted in the absence of any commercial or financial relationships that could be construed as a potential conflict of interest.

## Footnotes

1 Appendix H, *NeuroGuide Help Manual*, Thatcher, 2018.

2 The participants’ names have been changed to pseudonyms to maintain confidentiality and for ease of reference.

3 Done with Brainvision Analyzer Version 2.1.2.

## Appendix A

### Descriptions of Darryl’s Music Conditions for EEG Session

Own Pain music: 33 s duration; solo bell tree played with repetitive motif of single ascending notes (approximately F, F#, G#, A, returning to F and repeating) at approximately 30 beats/min. Gradually increasing in volume and intensity; timbre included ringing overtones with some dissonance.

Own Healing music: 84 s duration; strummed acoustic guitar played in 4/4 meter, approximately 112 beats/min; therapist’s female voice vocalizing “Ah” and “ooh” on melody of a Christian hymn in major key (“All Creatures of Our God and King”/ “*Lasst uns erfreuen herzlich sehr”* [using melody from an arrangement by Dearmer and Vaughan Williams, 1906]), with a bright sound.

Own Preferred music: 83 s duration; “St. Croix” from *The Sounds of the Caribbean* by Heintz and Higgins (2001a); ambient music; 4/4 meter, quarter note at approximately 84 beats per minute; soprano saxophone alternating with flute playing long descending phrases over textured electronic percussion and pedal tones, with treefrog song throughout.

Other Pain music: 42 s duration; thunder tube sounding in long phrases, gradually increasing in waves of sound, then diminishing in volume.

Other Healing music: 70 s duration; metallophone and voice together, C major tonality, repeating a melodic phrase with voice (on “oooh”) doubling metallophone on G above middle C and descending in long phrases of melody (each pitch dotted half-note length) from G to E, then F to D, and then E to middle C. Left hand played metallophone on harmonizing pitches underneath voice, playing on downbeat of each measure, with occasional passing quarter notes; 3/4 meter, quarter note approximately 76 beats/min.

### Descriptions of Will’s Music Conditions for EEG Session

Personal pain music: 43 s duration; glockenspiel played with repeated descending scalar motif (major mode, often tonic to dominant, occasionally subdominant to median) in upper range in quarter notes (4/4-like meter) with accompanying repeated single notes in left hand on dominant or other notes on beats 1 and 3 of measure; sound had occasional dissonance and sharp timbre; approximate tempo of quarter note 90 beats/min.

Personal Healing music: 87 s duration; metallophone played in lower range, with slower tempo than Pain music; more freeform, unclear meter at first, but settled into 4/4 meter after several seconds; repeated short melodic motif in higher register with short intervallic leaps mixed with quarter notes, accompanied with drone-like lower notes on downbeats (1 and 3 of measure) often on tonic; melody had major tonality; approximate tempo of quarter note 45 beats/minute.

Preferred music: 76 s duration; “Rain Forest” (Heintz and Higgins, 2001b): chirping birds; soprano sax playing melodic phrases in major key over piano in light jazz style; bass guitar countermelody underneath; pulse approximately 70 beats per minute.

### Descriptions of Carolyn’s Music Conditions for EEG Session

Personal pain music: 30 s in duration; solo cowbell played at steady pulse, at first at moderate volume, then gradually softer at 92 beats/min.

Personal healing music: 73 s in duration; soft volume electric keyboard, improvised scalar passages with phrases ending in chords in treble range with arpeggiated chords in bass, major tonality, 4/4 meter, soft volume with very gradually increasing volume about 2/3 through; quarter note approximately at 100 beats/min.

Preferred music: 76 s duration; Native American flute, playing an arrangement of the hymn Amazing Grace (Nakai, 2004); played freely in 3/4 meter at quarter note approximately 60 bpm; String ensemble playing sustained chords and harmonies underneath solo Native American flute; melody of the popular Christian hymn; major mode, occasional trading melodic role between flute and strings.
