# Erratum: Neuronal Effects of Listening to Entrainment Music Versus Preferred Music in Patients With Chronic Cancer Pain as Measured via EEG and LORETA Imaging

**DOI:** 10.3389/fpsyg.2022.866996

**Published:** 2022-02-22

**Authors:** Frontiers Production Office

**Affiliations:** Frontiers Media SA, Lausanne, Switzerland

**Keywords:** music therapy, EEG, LORETA (Low Resolution Electromagnetic Tomography), case study, chronic pain, cancer, pain

Due to a production error, a reference was wrongly included in the reference list and incorporated an incorrect DOI. The reference “Dileo, C., Hunt, A., Vetri, R., Raffa, R. B., and Rupnow-Kidd. (2021). Effects of music therapy entrainment on pain, vital signs, and bowel function of cancer patients. *Complement. Ther. Med*. 23, 714–718. doi: 10.1016/j.ctim.2015.08.002” should be removed from the reference list, and the in-text citation “Dileo et al., 2021” should instead be written as “Dileo et al., in preparation.”

The publisher apologizes for this mistake. The original article has been updated.

